# From emotion regulation to moral judgment: culturally responsive education and the psychological pathways of youth moral reasoning

**DOI:** 10.3389/fpsyg.2026.1869436

**Published:** 2026-06-18

**Authors:** Wenqi Ma, Juan Yang

**Affiliations:** 1 Faculty of Education, Northeast Normal University, Changchun, Jilin, China; 2 College of Intelligent Construction and Manufacturing, Sichuan Technology and Business University, Chengdu, China

**Keywords:** Chinese education, culturally responsive education, empathy, grade 12 students, moral reasoning, responsible decision-making, social–emotional competence

## Abstract

Culturally responsive education (CRE) is increasingly discussed as an inclusive pedagogical orientation, yet its relationship with adolescents’ moral reasoning remains underexplored. The present study examined whether CRE was associated with Grade 12 students’ moral reasoning through differentiated dimensions of social–emotional competence (SEC), with particular attention to emotion regulation, empathy and perspective taking, and responsible decision-making. Participants were 500 Chinese senior high school students (46.6% male, 53.4% female; M_age = 18.38 years, SD = 0.49) from two schools. Students completed measures of CRE, SEC, and scenario-based moral reasoning. Psychometric analyses supported the measurement structure, and the nine-factor CFA showed excellent fit, *χ*^2^(558) = 562.64, *p* = 0.437, CFI = 0.999, TLI = 0.999, RMSEA = 0.004, SRMR = 0.028. In the structural model, CRE was positively associated with emotion regulation (*β* = 0.284, *p* < 0.001), empathy and perspective taking (*β* = 0.465, *p* < 0.001), and responsible decision-making (*β* = 0.534, *p* < 0.001). Empathy and perspective taking (*β* = 0.309, *p* < 0.001) and responsible decision-making (*β* = 0.257, *p* < 0.001), but not emotion regulation (*β* = 0.027, *p* = 0.522), were positively associated with moral reasoning. The direct CRE to moral reasoning path was not significant after the SEC dimensions were included (*β* = 0.052, *p* = 0.287). Bootstrap analyses indicated significant indirect effects through empathy and perspective taking and responsible decision-making, but not through emotion regulation. The findings are consistent with an indirect-only pattern in which culturally responsive schooling is linked to moral reasoning primarily through specific socio-emotional pathways. The study extends work on culturally responsive pedagogy by showing that its relevance may include not only engagement and belonging, but also the psychological processes through which late adolescents reason about fairness, responsibility, and competing social claims.

## 1 Introduction

Late adolescence is a particularly important period for examining moral reasoning because it marks a shift from externally guided rule following toward more autonomous and reflective forms of value judgment (Eisenberg et al., 2005). For students aged 18 to 19, questions of fairness, harm, responsibility, group obligation, and self-positioning become more difficult as they approach the transition out of secondary schooling and into a wider social world (Kuther and Higgins-D'Alessandro, 2000). Moral reasoning at this stage is therefore not simply a matter of identifying what is right or wrong. It involves weighing competing claims under conditions of ambiguity, peer comparison, institutional pressure, and developmental change (Bajovic and Rizzo, 2021). These issues are especially salient in Grade 12. In the Chinese Gaokao context, students encounter intensified performance pressure, tightly regulated classroom routines, and heightened sensitivity to fairness, responsibility, and collective norms. School is thus not only a place where academic knowledge is transmitted, but also a setting in which students learn how to interpret rules, negotiate differences, and position themselves in relation to others and to wider social expectations (Collie et al., 2012). In educational environments that serve students from varied regional, socioeconomic, and ethnic backgrounds, students’ perceptions of whether their backgrounds and lived experiences are recognized may shape how they understand authority, belonging, and legitimacy within school life (Whitaker and Valtierra, 2018). From this perspective, culturally responsive education has potential relevance not only for engagement or inclusion, but also for the formation of value-based judgment (Comstock et al., 2023).

One plausible pathway linking culturally responsive education to moral reasoning runs through social–emotional competence. Social–emotional learning is not limited to emotional management training, but refers to a broader developmental process through which students learn to regulate emotions, understand others, build relationships, and make responsible decisions (Durlak et al., 2011; Schonert-Reichl, 2017). The present study focuses on three dimensions that are especially relevant to late-adolescent moral judgment: emotion regulation, empathy and perspective taking, and responsible decision-making (Lawson et al., 2019). These dimensions are unlikely to matter in identical ways. Emotion regulation may help adolescents manage conflict and inhibit impulsive reactions, whereas empathy and perspective taking and responsible decision-making are more directly involved in understanding competing standpoints and evaluating what ought to be done in morally ambiguous situations. Existing studies have linked perspective taking and empathic concern to prosocial and justice-related judgment, and school-based SEL research has shown that socio-emotional competencies are shaped by educational environments (Gülseven et al., 2020; Rasmussen et al., 2018; Coelho et al., 2015; Portela-Pino et al., 2024). Recent network-based work further suggests that emotion regulation is embedded within a broader SEC system rather than operating in isolation (Chen et al., 2025). What is still rarely tested, however, is a single explanatory model connecting culturally responsive education to differentiated SEC dimensions and then to moral reasoning. This gap is especially notable among Grade 12 students, whose socio-emotional and moral judgments develop under the demanding mix of developmental transition and examination pressure. The present study addresses that gap by examining whether students who perceive stronger culturally responsive education also report stronger socio-emotional competence and, in turn, stronger moral reasoning.

## 2 Literature review

### 2.1 Culturally responsive education and youth development

Culturally responsive education is best understood not as the simple addition of cultural content to classroom instruction, but as an educational orientation that connects teaching, participation, and school expectations to students’ backgrounds, lived experiences, and ways of meaning-making (Ladson-Billings, 1995; Comstock et al., 2023). Culture, in this sense, is not peripheral to learning. It forms part of the framework through which students interpret knowledge, authority, and belonging in school. Recent work has moved this discussion beyond normative advocacy by showing that culturally responsive teaching can be observed in concrete classroom practices and measurable instructional dispositions (Comstock et al., 2023; Franco et al., 2024). For adolescents, this matters because the school environment is not merely instructional. It is also developmental. Students are negotiating identity, legitimacy, participation, and social positioning while they learn. In Grade 12, these developmental issues become sharper because educational stakes are unusually high and opportunities for meaningful participation can narrow under exam pressure.

The developmental relevance of CRE becomes clearer when the relational structure of schooling is taken seriously. Earlier work on culturally responsive classroom management argued that participation, trust, and engagement are inseparable from the cultural norms embedded in teacher-student interaction (Weinstein et al., 2004). More recent evidence links culturally responsive teaching to school belongingness, teacher-student connectedness, and culturally relevant forms of support, all of which are closely tied to adolescents’ broader developmental functioning (Ialuna et al., 2024; Hernández et al., 2025; Chung et al., 2025; Morris, 2026). School-wide intervention studies also suggest that stronger cultural responsivity can improve classroom support and student engagement, indicating that CRE may operate as a meaningful developmental context rather than merely a symbolic commitment to diversity (Debnam et al., 2024). For Grade 12 students, such relational conditions may be especially consequential. When students experience school as a place where their voices and backgrounds are taken seriously, they may be more willing to engage reflectively, more likely to see institutional expectations as legitimate, and better positioned to develop the socio-emotional capacities that are relevant to morally charged decision making.

### 2.2 Social–emotional competence in educational contexts

Social–emotional competence refers to a set of capacities through which students manage emotions, understand others, maintain relationships, and make constructive decisions in social contexts. Current research treats these capacities not as secondary to schooling, but as part of the developmental foundation for participation, adjustment, and learning (Lawson et al., 2019; Kim et al., 2024). The present study narrows that broader framework to three dimensions that are particularly relevant to moral reasoning: emotion regulation, empathy and perspective taking, and responsible decision-making. This narrower focus matters because these dimensions differ in how directly they relate to moral judgment. Some function more as distal resources for self-management, whereas others are more proximal to how adolescents interpret and resolve moral conflict.

Emotion regulation refers to the ability to manage tension, delay impulsive reactions, and maintain reflective control under pressure. In school settings, this capacity may help students respond more adaptively to conflict, authority, and emotionally charged situations, and recent reviews suggest that it can be strengthened through intentional developmental supports (Lancastle et al., 2024). Even so, emotion regulation is not identical to moral judgment. It may help students stay calm enough to reflect, but it does not by itself orient them toward others’ perspectives or toward balancing fairness, care, rules, and responsibility. Empathy and perspective taking, by contrast, involve recognizing others’ feelings, circumstances, and standpoints. Responsible decision-making concerns how students weigh consequences, obligations, and competing values when deciding what ought to be done. Recent work also treats decision-making itself as an explicit instructional target within social–emotional learning (Moore and Gregory, 2024). These latter dimensions are therefore more proximal to moral reasoning itself. This proximal-distal distinction is central to the present study because it helps explain why differentiated SEC dimensions may show different patterns of association with moral reasoning.

### 2.3 Moral reasoning in late adolescence

Moral reasoning in late adolescence deserves particular attention because this period marks a transition from externally guided rule following toward more autonomous, reflective, and context-sensitive judgment. Recent work emphasizes that adolescents are not simply internalizing fixed moral rules. They are increasingly coordinating competing concerns such as fairness, welfare, rights, loyalty, and social expectations when evaluating complex situations (Malti et al., 2021; Killen and Dahl, 2021). This makes moral reasoning especially relevant for students aged 18 to 19, who stand at the threshold between adolescence and early adulthood while also navigating the academic demands and social pressures of Grade 12. In school life, these tensions are rarely abstract. They emerge in dilemmas involving cheating, exclusion, public blame, unequal treatment, and the conflict between institutional rules and contextual realities.

This developmental pattern is closely tied to ongoing changes in social cognition and perspective taking. As adolescents become better able to interpret interpersonal situations from multiple viewpoints, their moral judgments can also become more nuanced (Hollarek and Lee, 2022). Recent studies similarly suggest that adolescent moral judgment is shaped by relational and contextual variables, including attachment quality, empathy, and the kinds of conflict adolescents are asked to resolve (Ricon and Leopold Katz, 2024; Shao et al., 2024). Emerging work also links moral reasoning to adolescent decision processes under immersive social conflict and to prosocial civic decision-making, further underscoring the judgment-oriented nature of these competencies (Zhang and Zhang, 2025; Castro et al., 2025). Intervention evidence points in the same direction: moral reasoning can be strengthened when students have opportunities to discuss dilemmas, reflect on competing values, and practice ethical evaluation rather than simply receiving rule-based instruction (Bulut et al., 2024). For Grade 12 students, this issue has particular educational relevance because high-stakes schooling may heighten attention to fairness, discipline, and collective responsibility while at the same time narrowing opportunities for open discussion. School-based moral reasoning is therefore likely to remain sensitive to the broader classroom environment.

### 2.4 Integrating the three constructs

Bringing these strands of literature together suggests a coherent explanatory model. CRE may be relevant to moral reasoning not because it directly supplies moral conclusions, but because it shapes the social and psychological conditions under which students interpret difference, negotiate norms, and evaluate competing claims. Educational environments that recognize students’ backgrounds, respect their lived experiences, and encourage meaningful participation may strengthen legitimacy, reduce defensiveness, and widen the space for developmental growth. Within such environments, students may become better able to understand others’ perspectives, regulate their responses, and make judgments that take both interpersonal and social consequences into account. This logic supports a mediation framework. CRE can be treated as the developmental context, differentiated SEC dimensions as the proximal psychological pathways, and moral reasoning as the higher-order evaluative outcome. It also justifies the expectation that mediation may be substantial and that the more judgment-focused SEC dimensions may account for a larger share of the observed association than emotion regulation.

Building on the developmental picture established in the preceding sections, culturally responsive education, social–emotional competence, and moral reasoning can be understood as forming a sequential developmental pathway in which educational conditions are linked to the psychological resources through which moral judgment becomes possible. This integrative perspective also helps clarify why the present study does not treat social–emotional competence as a single undifferentiated mechanism. Emotion regulation may enable adolescents to remain reflective rather than impulsive in morally charged situations, but empathy and perspective taking and responsible decision-making are even more directly connected to how moral conflicts are interpreted and resolved. It is therefore reasonable to expect that culturally responsive education will be positively associated with students’ social–emotional competence, that social–emotional competence will in turn be positively associated with moral reasoning, and that culturally responsive education may also show a positive bivariate association with moral reasoning. At the same time, it is possible that the CRE-moral reasoning association operates primarily through socio-emotional development rather than through a direct pathway, in which case an indirect-only pattern would be theoretically plausible. Consistent with this logic, social–emotional competence is expected to mediate the association between culturally responsive education and high school students’ moral reasoning.

H1, culturally responsive education is positively associated with high school students’ social–emotional competence;

H2, social–emotional competence is positively associated with high school students’ moral reasoning, with empathy and perspective taking and responsible decision-making expected to show stronger associations than emotion regulation;

H3, culturally responsive education is positively associated with high school students’ moral reasoning;

H4, social–emotional competence mediates the relationship between culturally responsive education and high school students’ moral reasoning.

## 3 Methodology

### 3.1 Research design

This study used a cross-sectional survey design to examine whether students’ perceptions of culturally responsive education (CRE) were associated with moral reasoning and whether this association was linked to differentiated dimensions of social–emotional competence (SEC). The analytic model positioned CRE as the focal educational-context variable, three SEC dimensions as mediating processes, and moral reasoning as the outcome construct. Because the data were collected at a single time point, the study does not make causal claims. Instead, the analyses clarify how these constructs were linked within the lived school experience of late-adolescent students preparing for the National College Entrance Examination (Gaokao), a context in which classroom recognition, fairness, and cultural legitimacy may be especially relevant to how students regulate emotion, interpret others’ perspectives, and reason through moral dilemmas.

### 3.2 Participants and procedure

The final analytic sample consisted of 500 Grade 12 students from two senior high schools in China. One school was a general senior high school and the other was classified as a key senior high school. Of the participants, 233 were male (46.6%) and 267 were female (53.4%). Ages ranged from 18 to 19 years (M = 18.38, SD = 0.49). In addition, 55.6% of the sample reported an urban background and 44.4% reported a rural background. Questionnaires were administered during regular class periods in a supervised classroom setting after coordination with school administrators and homeroom teachers. Data collection was completed at the time arranged by each school, and all students in the same class completed the questionnaire under the same conditions. The survey was administered in paper and pencil format, and completion took approximately 15 to 20 min. Homeroom teachers helped maintain normal classroom order but did not explain questionnaire items, inspect responses, or intervene in students’ answers. Students were seated separately where possible and were instructed to complete the questionnaire independently without discussion with classmates. Students were informed that participation was voluntary, responses would remain anonymous, and the data would be used only for research purposes. Questionnaires were checked after collection for item completeness, patterned responding, duplicate responses, and obviously invalid answer sheets. A small number of students were absent or did not return usable questionnaires, and these cases were not included in the final analytic dataset. No returned questionnaire included item level missing values after screening, and no case met the exclusion criteria for invalid responding. Therefore, all 500 valid returned questionnaires were retained for analysis. Because there were no item level missing values in the retained dataset, no imputation procedure was required.

### 3.3 Measures

The survey package included measures of CRE, SEC, and moral reasoning, along with demographic and family-background variables. Unless otherwise noted, items were rated on a five-point Likert scale from 1 (strongly disagree) to 5 (strongly agree). CRE was operationalized through three lower-order dimensions: cultural respect and identity recognition, connection between instruction and lived experience, and climate of diverse expression and fair interaction. Item development drew conceptually on culturally responsive pedagogy and student-perception research and was contextually adapted for Chinese senior high school classrooms. A representative item was: “Teachers respect students from different family or regional backgrounds.” SEC was measured with reference to CASEL-informed literature, but the instrument focused only on the three dimensions most relevant to the present model: emotion regulation, empathy and perspective taking, and responsible decision-making (Lawson et al., 2019). Example items included: “I can calm myself when I feel upset or misunderstood,” “I try to look at a situation from another student’s point of view,” and “Before making an important decision, I think about how it may affect others.” Moral reasoning was assessed using a scenario-based instrument rather than a purely abstract self-report scale. Four school-relevant dilemmas were developed to reflect conflicts plausible in late-adolescent educational settings, including academic dishonesty under examination pressure, conflict between school rules and students’ family or community practices, exclusion of lower-performing classmates for collective academic advantage, and online condemnation of a student who had made a mistake. The scenarios were reviewed for contextual realism and developmental appropriateness and were pilot-tested for wording clarity, comprehension, and completion time. For each scenario, students rated the importance of response considerations related to fairness and care orientation, integration of rules and responsibility, and reasoning complexity. These ratings were aggregated into dimension scores and an overall moral reasoning index. Cronbach’s *α* values ranged from 0.820 to 0.858 across the nine lower-order dimensions.

### 3.4 Indicator structure and aggregation logic

The measurement model was organized at two levels. At the lower-order level, the study included nine dimensions: three CRE dimensions, three SEC dimensions, and three moral reasoning dimensions. These lower-order dimensions were used in the reliability and validity analyses to demonstrate psychometric adequacy. At the higher-order analytic level, the CRE dimensions were aggregated into an overall CRE score, the three SEC dimensions were retained as differentiated mediators, and the three moral reasoning dimensions were aggregated into an overall moral reasoning index. This two-level structure explains why the psychometric tables report nine dimensions whereas the structural results focus on five broader constructs.

### 3.5 Data analysis

All analyses were conducted in R 4.3.1. The analytic sequence followed six steps. Step 1 involved descriptive analysis of the demographic variables and the major constructs, along with zero-order correlations and distributional checks. Step 2 evaluated reliability and validity using Cronbach’s *α*, composite reliability (CR), average variance extracted (AVE), the Fornell–Larcker criterion, and HTMT ratios. Step 3 estimated a nine-factor confirmatory factor analysis (CFA) to test the lower-order measurement structure; model fit was evaluated using chi-square divided by degrees of freedom, CFI, TLI, RMSEA, and SRMR. Step 4 estimated the structural model using aggregated construct scores, with CRE as the predictor, the three SEC dimensions as mediators, and moral reasoning as the outcome. Step 5 examined indirect effects using 5,000 bootstrap resamples and 95% confidence intervals. Step 6 assessed common method bias with Harman’s single-factor test. As a robustness check, the main path pattern was also re-estimated with demographic covariates, and the substantive conclusions remained unchanged.

### 3.6 Control variables

Several background variables were included as controls to reduce the possibility that the observed relationships were driven by broader demographic or family conditions rather than by the focal constructs. These variables included gender, exact age, urban–rural background, parental education, perceived family socioeconomic status, school type, and self-rated academic performance. Such controls were included because moral reasoning and socio-emotional development are shaped not only by school experience but also by family resources, social context, and prior educational opportunity (Table 1).

**Table 1:** Indicator system of the study variables.

Construct	Dimension	Operational meaning	Sample indicators	Suggested items
Culturally responsive education	Cultural respect and identity recognition	The extent to which students feel that their backgrounds, identities, and lived experiences are respected and recognized in school	Teachers respect different family and regional backgrounds; students feel their experiences are understood; no single cultural experience is treated as the only valid one	4
Culturally responsive education	Connection between instruction and lived experience	The extent to which teaching content is meaningfully connected to students’ daily lives, communities, and real social issues	Classroom examples are related to students’ real life; lessons help students understand issues around them; school knowledge feels relevant to actual experience	4
Culturally responsive education	Climate of diverse expression and fair interaction	The extent to which classroom interaction is inclusive, fair, and open to different viewpoints	Different opinions can be expressed safely; students from different backgrounds are treated fairly; classroom discussion encourages genuine participation	4
Social–emotional competence	Emotion regulation	The ability to manage negative emotions, inhibit impulsive reactions, and remain reflective under pressure or conflict	Students can calm themselves when upset; they do not react impulsively when misunderstood; they can regulate emotions in stressful situations	4
Social–emotional competence	Empathy and perspective taking	The ability to understand others’ feelings, positions, and circumstances	Students try to think from another person’s point of view; they can sense classmates’ emotional difficulties; they are willing to understand reasons behind others’ actions	4
Social–emotional competence	Responsible decision-making	The ability to weigh fairness, rules, consequences, relationships, and responsibility when making judgments	Students consider whether decisions may harm others; they think about long-term consequences; they try to balance rules and real circumstances	4
Moral reasoning	Fairness and care orientation	The extent to which moral judgments take into account others’ rights, harm, and well-being	Attention to fairness, harm prevention, and concern for others in scenario responses	4 scenario ratings
Moral reasoning	Integration of rules and responsibility	The extent to which students balance institutional rules with contextual realities and moral responsibility	Judgments reflect both norm compliance and contextual understanding rather than rigid obedience	4 scenario ratings
Moral reasoning	Reasoning complexity	The extent to which students make multi-perspective, context-sensitive, and principled judgments when facing moral dilemmas	Responses reflect the ability to consider multiple viewpoints, integrate competing values, and justify decisions beyond a single rule or immediate interest	4 scenario ratings

All items for culturally responsive education and social–emotional competence were measured on a five-point Likert scale ranging from 1 = strongly disagree to 5 = strongly agree. Moral reasoning was assessed through four school-based dilemma scenarios, and scenario scores were aggregated into three evaluative dimensions and then combined into an overall index. Lower-order dimensions were used for psychometric evaluation and later aggregated into higher-order constructs for the structural analyses.

## 4 Results

### 4.1 Psychometric evaluation of the measurement model

The psychometric analyses were conducted at the level of the nine lower-order dimensions underlying the broader study constructs. Internal consistency was acceptable to good across all dimensions, with Cronbach’s *α* values ranging from 0.820 to 0.858. Composite reliability values ranged from 0.820 to 0.857, and AVE values ranged from 0.533 to 0.601, indicating acceptable convergent validity across the CRE, SEC, and moral reasoning dimensions. Although these values are fairly tidy, this pattern is not unexpected given that each lower-order dimension consisted of four parallel indicators and was developed as a short, internally coherent subscale (Tables 2, 3).

**Table 2:** CFA model fit indices for the nine-factor measurement model.

Model	*χ* ^2^	df	*p*	CFI	TLI	RMSEA	SRMR
Nine-factor measurement model	562.64	558	0.437	0.999	0.999	0.004	0.028

Conventional interpretive guidelines suggest that lower *χ*^2^/df values and higher CFI/TLI values indicate better fit, whereas lower RMSEA and SRMR values indicate less residual misfit. These thresholds were treated as descriptive guides rather than rigid decision rules.

**Table 3:** Reliability and convergent validity.

Construct	Dimension	Items	Cronbach’s *α*	CR	AVE
Culturally responsive education	Cultural respect and identity recognition	4	0.841	0.841	0.569
Culturally responsive education	Connection between instruction and lived experience	4	0.858	0.857	0.601
Culturally responsive education	Climate of diverse expression and fair interaction	4	0.829	0.829	0.547
Social–emotional competence	Emotion regulation	4	0.836	0.836	0.561
Social–emotional competence	Empathy and perspective taking	4	0.857	0.856	0.598
Social–emotional competence	Responsible decision-making	4	0.838	0.837	0.563
Moral reasoning	Fairness and care orientation	4	0.848	0.848	0.582
Moral reasoning	Integration of rules and responsibility	4	0.826	0.826	0.542
Moral reasoning	Reasoning complexity	4	0.820	0.820	0.533

A nine-factor CFA was estimated to test the lower-order measurement structure prior to the structural analyses. The model fit the data very well, *χ*^2^(558) = 562.64, *p* = 0.437, CFI = 0.999, TLI = 0.999, RMSEA = 0.004, SRMR = 0.028. These values indicate that the lower-order dimensions were empirically distinguishable. At the same time, this excellent fit should not be over-interpreted as evidence of a flawless instrument. The model consisted of short, conceptually narrow four-item subscales and scenario-based ratings, a structure that can yield very strong global fit when indicators are well aligned with their intended dimensions (Table 4).

**Table 4:** Fornell–Larcker criterion.

Construct	1	2	3	4	5
Culturally responsive education	**0.653**	0.284	0.465	0.534	0.334
Emotion regulation	0.284	**0.749**	0.186	0.171	0.140
Empathy and perspective taking	0.465	0.186	**0.773**	0.473	0.451
Responsible decision-making	0.534	0.171	0.473	**0.750**	0.427
Moral reasoning	0.334	0.140	0.451	0.427	**0.745**

Diagonal values in bold are the square roots of AVE.

Discriminant validity was supported by two criteria. First, the square roots of the AVE values exceeded the corresponding inter-construct correlations in the Fornell–Larcker matrix. Second, all HTMT ratios remained below the conventional 0.85 threshold. The highest HTMT value was observed between CRE and responsible decision-making, which is theoretically plausible because both are tied to participatory and context-sensitive aspects of school life, yet the value remained below the threshold indicating problematic overlap (Table 5).

**Table 5:** HTMT ratios.

Construct	1	2	3	4	5
Culturally responsive education	–				
Emotion regulation	0.328	–			
Empathy and perspective taking	0.529	0.220	–		
Responsible decision-making	0.615	0.204	0.558	–	
Moral reasoning	0.364	0.160	0.503	0.482	–

At the higher-order construct level, the mean scores ranged from 3.398 to 3.570, indicating moderate to moderately positive levels of CRE, SEC, and moral reasoning in the sample. Zero-order correlations were consistent with the proposed model. CRE was positively related to emotion regulation (*r* = 0.284, *p* < 0.001), empathy and perspective taking (*r* = 0.465, *p* < 0.001), responsible decision-making (*r* = 0.534, *p* < 0.001), and moral reasoning (*r* = 0.334, *p* < 0.001). Among the SEC dimensions, empathy and perspective taking (*r* = 0.451, *p* < 0.001) and responsible decision-making (*r* = 0.427, *p* < 0.001) showed stronger links to moral reasoning than emotion regulation (*r* = 0.140, *p* < 0.01). Harman’s single-factor test further showed that the first unrotated factor accounted for 30.82% of the total variance, suggesting that common method bias was unlikely to fully account for the observed pattern of associations.

### 4.2 Descriptive statistics and correlations among the main variables

Descriptive statistics and correlations shown in Table 6.

**Table 6:** Descriptive statistics and correlations.

Variable	M	SD	1	2	3	4	5
Culturally responsive education	3.489	0.506	–				
Emotion regulation	3.398	0.600	0.284***	–			
Empathy and perspective taking	3.570	0.608	0.465***	0.186***	–		
Responsible decision-making	3.518	0.605	0.534***	0.171***	0.473***	–	
Moral reasoning	3.509	0.561	0.334***	0.140**	0.451***	0.427***	–

***p < 0.001.

### 4.3 structural path analysis

The structural association model was estimated on the aggregated higher-order constructs. CRE was positively associated with all three SEC dimensions: emotion regulation (*β* = 0.284, *p* < 0.001), empathy and perspective taking (*β* = 0.465, *p* < 0.001), and responsible decision-making (*β* = 0.534, *p* < 0.001). The SEC dimensions, however, did not contribute equally to moral reasoning. Emotion regulation was not significantly associated with moral reasoning once the other SEC dimensions were included (*β* = 0.027, *p* = 0.522). By contrast, empathy and perspective taking (*β* = 0.309, *p* < 0.001) and responsible decision-making (*β* = 0.257, *p* < 0.001) were both positively associated with moral reasoning. The direct CRE to moral reasoning path was small and not statistically significant after the mediators were included (*β* = 0.052, *p* = 0.287). This pattern is consistent with an indirect-only or primarily indirect structural pattern rather than a strong remaining direct pathway. Robustness checks that included demographic covariates yielded the same substantive pattern (Table 7).

**Table 7:** Structural association estimates.

Path	Std. *β*	SE	*z*	*p*	Interpretation
CRE → Emotion regulation	0.284	0.054	6.213	<0.001	Significant
CRE → Empathy and perspective taking	0.465	0.048	11.515	<0.001	Significant
CRE → Responsible decision-making	0.534	0.044	14.588	<0.001	Significant
Emotion regulation → moral reasoning	0.027	0.038	0.640	0.522	ns
Empathy and perspective taking → moral reasoning	0.309	0.039	7.269	<0.001	Significant
Responsible decision-making → moral reasoning	0.257	0.043	5.375	<0.001	Significant
CRE → Moral reasoning	0.052	0.053	1.065	0.287	ns

### 4.4 Mediation analysis

Bootstrap analyses provided a more differentiated test of H4. The indirect pathway through emotion regulation was not significant (indirect estimate = 0.008, 95% CI [−0.017, 0.035]). By contrast, the indirect pathway through empathy and perspective taking was significant (indirect estimate = 0.156, 95% CI [0.109, 0.207]), as was the indirect pathway through responsible decision-making (indirect estimate = 0.149, 95% CI [0.092, 0.211]). The total indirect estimate was also significant (0.314, 95% CI [0.240, 0.392]). These results indicate that H4 was partially supported overall. CRE was associated with moral reasoning primarily through two specific socio-emotional pathways, not through SEC as a fully uniform mechanism. The pattern is therefore better described as consistent with an indirect-only pathway than as a complete confirmation of full mediation in a strong causal sense (Table 8).

**Table 8:** Bootstrap estimates of indirect associations.

Indirect path	Effect	Boot SE	95% CI lower	95% CI upper	Result
CRE → Emotion regulation → moral reasoning	0.008	0.013	−0.017	0.035	Not supported
CRE → Empathy and perspective taking → moral reasoning	0.156	0.025	0.109	0.207	Supported
CRE → Responsible decision-making → moral reasoning	0.149	0.030	0.092	0.211	Supported
Total indirect effect	0.314	0.039	0.240	0.392	Supported

### 4.5 Summary of hypothesis testing

Taken together, the hypothesis tests suggest that CRE was positively associated with students’ social–emotional competence, but the SEC dimensions did not operate uniformly. Empathy and perspective taking and responsible decision-making showed clear positive links to moral reasoning, whereas emotion regulation did not. The direct CRE to moral reasoning path was not significant after the mediators were included. Accordingly, H1 was supported, H2 was partially supported, H3 was not supported, and H4 was partially supported. The overall pattern indicates that CRE was associated with moral reasoning primarily through selected indirect pathways rather than through a robust direct relationship (Table 9).

**Table 9:** Summary of hypothesis testing.

Hypothesis	Statement	Result
H1	Culturally responsive education positively predicts high school students’ social–emotional competence.	Supported
H2	Social–emotional competence positively predicts high school students’ moral reasoning.	Partially supported
H3	Culturally responsive education positively predicts high school students’ moral reasoning.	Not supported
H4	Social–emotional competence mediates the relationship between culturally responsive education and high school students’ moral reasoning.	Partially supported

### 4.6 Robustness text

Table 10 reports the covariate adjusted robustness model. After gender, age, urban rural background, father’s education, mother’s education, perceived family socioeconomic status, school type, and self rated academic performance were included, the main pattern remained stable. CRE was still positively associated with emotion regulation, empathy and perspective taking, and responsible decision making, indicating that students who perceived stronger culturally responsive education also reported higher levels of these socio emotional competencies even after background differences were considered. In the moral reasoning model, empathy and perspective taking and responsible decision making remained significant positive predictors, whereas emotion regulation remained non significant. The direct path from CRE to moral reasoning also remained non significant after covariate adjustment. These results support the robustness of the main interpretation: CRE was linked to moral reasoning mainly through empathy and perspective taking and responsible decision making, rather than through emotion regulation or a strong direct pathway. The adjusted model also reduces concern that the observed associations were merely artifacts of gender composition, family background, school type, or academic performance differences. Nevertheless, these findings should still be interpreted as robustness evidence within a cross sectional design, not as evidence of causal effects.

**Table 10:** Covariate-adjusted robustness model.

Path	Adjusted *β*	SE	*t*	*p*	Conclusion
CRE → Emotion regulation	0.282	0.044	6.478	<0.001	Robust
CRE → Empathy and perspective taking	0.45	0.04	11.268	<0.001	Robust
CRE → Responsible decision-making	0.528	0.038	13.751	<0.001	Robust
Emotion regulation → moral reasoning	0.033	0.04	0.817	0.414	Not significant, consistent with main model
Empathy and perspective taking → moral reasoning	0.293	0.046	6.412	<0.001	Robust
Responsible decision-making → moral reasoning	0.253	0.047	5.341	<0.001	Robust
CRE → Moral reasoning	0.043	0.048	0.889	0.375	Not significant, consistent with main model

## 5 Discussion

### 5.1 CRE as a developmental condition rather than a simple instructional style

The first major finding is that CRE was positively associated with all three SEC dimensions, although the strength of these associations differed. This matters because it suggests that culturally responsive education does more than make instruction feel relevant. For Grade 12 students, especially those facing the compressed and evaluatively intense Gaokao year, a classroom climate that acknowledges students’ backgrounds and treats their perspectives as legitimate may also shape how they interpret authority, participation, and interpersonal obligation. In other words, CRE appears to operate less like a narrow pedagogical technique and more like a developmental condition within school life.

The pattern across SEC dimensions is especially informative. CRE was most strongly associated with responsible decision-making, followed by empathy and perspective taking, while the association with emotion regulation was smaller. This ordering is theoretically coherent. A culturally responsive classroom often requires students to listen across differences, consider fairness in participation, and make sense of competing views. Those experiences are more directly aligned with judgment-oriented capacities than with basic emotional self-control. Emotion regulation is still relevant, but it appears to sit further upstream.

### 5.2 Why emotion regulation looked more distal

One of the more useful contributions of the study is the differentiated role of SEC. Emotion regulation showed only a weak zero-order correlation with moral reasoning and did not remain significant once empathy and perspective taking and responsible decision-making were entered into the model. This does not mean emotion regulation is unimportant for adolescent development. Rather, it suggests that in the present context it functions as a more distal resource. Under the high-pressure conditions of Grade 12 and Gaokao preparation, emotional management may be directed primarily toward academic self-regulation, stress tolerance, and performance maintenance rather than toward the evaluative work involved in moral judgment. Emotion regulation also appears likely to share variance with the more judgment-focused SEC dimensions. Once empathy and perspective taking and responsible decision-making are included in the same model, the portion of variance in moral reasoning uniquely attributable to emotional control may be substantially reduced.

By contrast, empathy and perspective taking and responsible decision-making were more proximal to moral reasoning. That result is psychologically plausible. School-based moral dilemmas rarely ask students only to calm themselves down; they ask students to understand who may be harmed, what counts as fair treatment, whether rules should be applied rigidly, and what responsibilities matter most in context. In a high-stakes Grade 12 environment, where academic competition and collective class pressure can intensify fairness concerns, these two dimensions are particularly likely to shape how students approach moral judgment. Recent calls for culturally adaptive models of empathy development are consistent with this interpretation, because they locate empathy within social meaning systems rather than treating it as a decontextualized trait (Sultan and Khan, 2025). Emotion regulation therefore appears to function as a more distal socio-emotional resource in this context, which aligns with theoretical expectations that judgment-focused competencies are more proximal drivers of moral reasoning.

### 5.3 Interpreting the indirect pattern cautiously

The structural results did not show a significant direct association between CRE and moral reasoning once the SEC mediators were included. At the same time, significant indirect pathways were observed through empathy and perspective taking and responsible decision-making. This is best interpreted as consistent with an indirect-only pattern, not as conclusive proof that CRE is linked to moral reasoning entirely through SEC. Given the cross-sectional design, strong causal language would overstate what the data can support.

Even with that caution, the pattern is theoretically meaningful. It suggests that culturally responsive educational experiences may matter for moral judgment less by directly teaching students what moral conclusion to reach and more by strengthening the interpersonal and evaluative capacities through which students reason about difficult situations. This interpretation is also more educationally realistic. Teachers do not typically improve moral reasoning by delivering moral answers. They more often shape the climate in which students learn to listen, consider multiple standpoints, and make responsible judgments under competing pressures. Recent work on culturally responsive connectedness and belonging reinforces this interpretation by showing that such classroom practices are closely tied to relational conditions that support reflective participation (Chung et al., 2025; Morris, 2026).

### 5.4 Limitations and future directions

Several limitations should be kept in view. The design was cross-sectional, so temporal ordering remains theoretical rather than demonstrated. The study also relied heavily on self-report measures, and although the common method checks did not indicate a dominant single-factor problem, shared method variance cannot be ruled out. The sample was drawn from only two schools, one general and one key senior high school, which limits the extent to which the observed pattern can be generalized to the broader national population of Chinese adolescents. The present findings are also embedded in the Gaokao context, and this high pressure educational culture may shape both socio-emotional functioning and moral judgment in ways that are not identical to lower-pressure school settings. The moral reasoning measure was scenario based. This strengthens contextual relevance but does not guarantee close correspondence with real-world moral behavior. In addition, the analyses did not model all plausible confounders, such as teacher level classroom variation, prior disciplinary experiences, or broader school climate variables beyond CRE. These limitations point to a clear research agenda. Future work would benefit from longitudinal and multi-wave designs, cross regional sampling within China, and cross-cultural comparison so that the generalizability of the present pattern can be tested more directly. Multi methods assessment, including teacher reports, peer reports, behavioral indicators, and qualitative dilemma discussions, would also help clarify how students’ reported moral reasoning corresponds to enacted moral behavior. The non-significant role of emotion regulation may also be context dependent, and its association with moral reasoning may look different in other age groups, educational stages, or cultural settings where academic pressure is organized differently.

## 6 Conclusion

This study examined whether culturally responsive education was associated with Grade 12 students’ moral reasoning and whether that association was linked to differentiated dimensions of social–emotional competence. The findings show that students who perceived their educational environment as more culturally responsive also reported stronger emotion regulation, empathy and perspective taking, and responsible decision-making. However, these socio-emotional dimensions did not contribute equally to moral reasoning. The more proximal links were found for empathy and perspective taking and responsible decision-making, whereas emotion regulation did not show an independent association once the other dimensions were considered. The broader implication is that culturally responsive education appears to be linked to moral reasoning primarily through indirect pathways rather than through a strong direct association. In the context of Chinese Grade 12 schooling, where fairness, responsibility, and social pressure are often intensified by high-stakes examination demands, these differentiated pathways are particularly meaningful. For practice, the findings suggest that teachers designing culturally responsive instruction should place particular emphasis on opportunities that strengthen empathy, perspective taking, and responsible decision-making, because these competencies were more closely associated with students’ moral judgment. School environments should also attend to students’ diverse backgrounds and maintain a fair, participatory classroom climate so that these indirect psychological pathways have room to develop. These implications are best understood as practical inferences from an observed pattern of associations rather than as proof of causal impact.

## Data Availability

The original contributions presented in the study are included in the article/supplementary material, further inquiries can be directed to the corresponding author.
